# Chorea-acanthocytosis presenting with epilepsy at onset harboring novel compound heterozygous *VPS13A* mutations

**DOI:** 10.1016/j.prdoa.2026.100433

**Published:** 2026-03-15

**Authors:** Mitsuyoshi Tamura, Shogo Furukawa, Masahiro Namiki, Yukiko Ozawa, Yuka Urata, Masayuki Nakamura, Mitsuru Watanabe, Hideki Oshima, Toshiki Obuchi, Tadashi Ichikawa, Yuriko Kikkawa

**Affiliations:** a Department of Neurology, Japanese Red Cross Narita Hospital, Chiba, Japan; b Department of Neurology, Graduate School of Medicine, Chiba University, Chiba, Japan; c Department of Psychiatry, Kagoshima University Graduate School of Medical and Dental Sciences, Kagoshima, Japan; d Department of Neurosurgery, Saitama Rehabilitation Center, Saitama, Japan; e Department of Neurological Surgery, Division of Neurosurgery, Nihon University School of Medicine, Tokyo, Japan; f Department of Neurology, Saitama Rehabilitation Center, Saitama, Japan

## 1 Introduction

Chorea-acanthocytosis (ChAc) is a rare neurodegenerative disorder marked by chorea, dystonia, cognitive impairment, epilepsy, neuropathy, and neuropsychiatric symptoms [1]. The initial symptoms of ChAc typically include involuntary movements and behavioral symptoms [2]. Elevated serum creatine kinase (hyperCKemia) and acanthocytes in peripheral blood smears are characteristic findings. The disease results from mutations in the vacuolar protein sorting 13 homolog A (*VPS13A*) gene on chromosome 9q21, therefore, partly due to its highly diverse clinical phenotype, it is now preferably summarized under the term VPS13A disease [1]. *VPS13A* mutations reduce chorein expression, leading to striatal degeneration and abnormal erythrocyte membranes [3]. Drug therapy is generally ineffective or provides partial relief for hyperkinetic/choreatic involuntary movements, while deep brain stimulation (DBS) may improve motor symptoms [4]. We describe a ChAc case without detectable acanthocytes that initially presented with epilepsy. We also report two novel *VPS13A* mutations and discuss the efficacy of globus pallidus internus (GPi) DBS.

A 25-year-old Japanese man with no prior medical history presented with a generalized tonic-clonic seizure, diagnosed as generalized epilepsy. Seizures recurred nearly yearly but were well controlled with levetiracetam (2,000 mg daily). At age 29, persistent hyperCKemia was detected. By age 32, he developed orolingual movements, tongue biting and tongue protrusion dystonia, causing feeding difficulty. At age 35, restlessness and wiggling-like limb movements were observed. He was diagnosed with mild intellectual disability and Tourette syndrome at a psychiatric hospital. Aripiprazole (6 mg) was ineffective. Progressive orofacial and limb movements impaired feeding and forced him to leave his job. As eating required assistance in a supine position, he was referred to our hospital at age 37. There was no family history of involuntary movements or epilepsy, although his separated father was reportedly diagnosed with unexplained hyperCKemia.

**Fig. 1:**
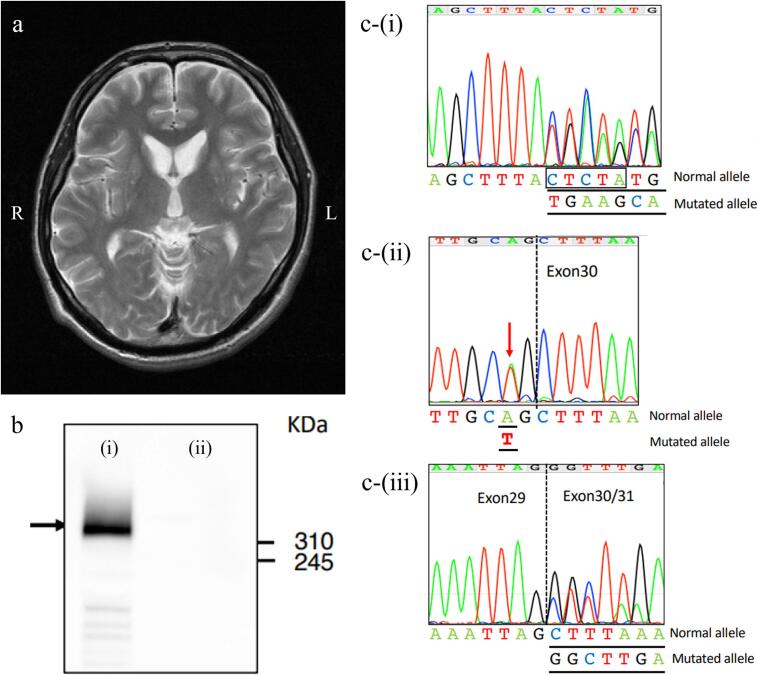
(a) Brain MRI at the first visit to our hospital. The T2-weighted image revealed atrophy of the bilateral caudate heads, while other brain regions appeared remarkably preserved. R, right side; L, left side. (b) Western blotting of chorein on erythrocyte membranes. The normal control is shown in (i). Chorein immunoreactivity was markedly reduced in the patient (ii). The black arrow indicates the chorein band. (c) Results of *VPS13A* genetic testing using Sanger sequencing. (i) In exon 16, a heterozygous c.1396_1400delCTCTCTA mutation was detected, resulting in a frameshift in which leucine is replaced by a stop codon (p.Leu466Ter) after deletion. (ii) Genomic DNA sequencing revealed a heterozygous c.3119-2A > T mutation at the splice acceptor site of intron 29. This mutation caused splicing out of exon 30, as described in the complementary DNA sequencing result (iii), resulting in a downstream stop codon.

He exhibited dysarthria, and involuntary orofacial movements appeared during conversation. Chorea was evident when the proximal limbs or trunk were held out or in use. While walking, foot dystonia with plantar extension was observed. To counteract the dystonia, he exaggerated leg elevation, resulting in gait imbalance (Video 1). Deep tendon reflexes were absent in all limbs. Laboratory tests revealed elevated creatine kinase (1,045 U/L; reference 60–287 U/L) and lactate dehydrogenase (253 U/L; reference 119–229 U/L). Brain magnetic resonance imaging showed bilateral striatal atrophy (Fig. 1a).

Although ChAc was strongly suspected based on clinical features, repeated peripheral blood smears revealed no acanthocytes. A western blot analysis of erythrocyte membranes showed markedly reduced chorein immunoreactivity (Fig. 1b). *VPS13A* genetic testing identified two novel heterozygous mutations: c.1396_1400delCTCTA in exon 16 and c.3119-2A > T in intron 29 (Fig. 1c). The exon 16 variant results in deletion of the 5-nucleotide sequence CTCTA, causing a frameshift mutation in which the codon for leucine (CTC) at position 466 is converted to a stop codon (TGA). The intron 29 variant affects a canonical splice acceptor site and is predicted to cause skipping of exon 30 (117 bp, a multiple of three), resulting in an in-frame deletion. The pathogenicity of both variants was supported by the complete absence of chorein on western blot analysis. Based on the biochemical and genetic findings, a diagnosis of ChAc was confirmed.

Haloperidol (6 mg), tiapride (300 mg), and levodopa/benserazide (300 mg) were administered sequentially to treat severe chorea and dystonia. However, pharmacologic therapy provided insufficient control. Given the patient's strong desire for improvement and reports suggesting a partial benefit of DBS in ChAc [4], bilateral GPi-DBS was considered. After detailed evaluation and informed consent, electrodes were implanted in the bilateral GPi using SenSight™ directional leads connected to a Percept™ PC neurostimulator (Medtronic). Under stimulation (bilateral: 60 μs, 130 Hz, 3.6 mA) and 100 mg of tiapride, involuntary movements—particularly foot dystonia—markedly improved, reducing gait disturbance substantially (Video 1). Upper-limb and tongue movements also improved substantially, allowing the patient to eat and drink independently.

In this case, epilepsy appeared as the initial symptom before progressive manifestations typical of ChAc. Coexisting epilepsy may help distinguish ChAc from phenotypically similar conditions, with the combination of choreatic movement disorder, elevated CK levels, and diminished/absent tendon reflexes constitutes the key phenotypic triad for diagnosing VPS13A disease. Previous reports have described epilepsy at onset as uncommon in ChAc [2]. However, in a Japanese cohort, seizure onset was not uncommon, occurring in 7 of 17 cases (or 7 of 16 when excluding cases with unknown onset symptoms), which is even more frequent than orofacial involuntary movement (4 cases) or chorea (2 cases) [1]. Therefore, seizure onset should not be regarded as atypical in ChAc. In this patient, acanthocytes—typically a key diagnostic feature—were not detected on repeated peripheral blood smears. Acanthocytosis may be undetectable early in the disease course; in this case, it remained absent even after 12 years from onset. Despite this, genetic testing identified pathogenic *VPS13A* variants, providing a definitive diagnosis. Therefore, even when acanthocytes are not observed using conventional smears, genetic testing should be performed in patients with characteristic clinical features of ChAc. Furthermore, because acanthocytes are best visualized in fresh wet preparations, they may be missed in conventional dried or fixed smears, and a false-negative finding cannot be excluded in this case.

ChAc exhibits considerable phenotypic variability, and genetic heterogeneity likely contributes to this diversity. More than half of Japanese patients with ChAc carry the exon 37 4411C > T (R1471X) mutation or exon 60–61 deletion and follow a relatively uniform clinical course [1]. Additionally, patients with the c.2343del mutation often present with epilepsy [2]. However, the genotype–phenotype relationship remains poorly defined. Given the wide spectrum of *VPS13A* variants, further studies are warranted.

Another key finding is the favorable response of drug-resistant involuntary movements to GPi-DBS. Conventional pharmacologic options—including levodopa, benzodiazepines, and baclofen—generally provide limited benefit for chorea and dystonia in ChAc [5]. In this patient, GPi-DBS markedly alleviated orofacial and limb dystonia/chorea, improving gait and restoring independent eating. Although reports on DBS for ChAc remain limited, they are increasing. Notably, in a study of 20 patients who underwent GPi-DBS, 15 demonstrated approximately 25% improvement in the Unified Huntington's Disease Rating Scale–motor score [4]. Also, it was noteworthy that DBS improved feeding difficulties in our case. While DBS is typically less effective for dysarthria and dysphagia, several reports describe a significant benefit in selected ChAc cases [5]. Therefore, DBS may represent an effective therapeutic strategy for drug-resistant involuntary movements. This patient maintained clinical stability for more than 2 years post-surgery.

We report a case of ChAc that initially presented with epilepsy, followed by mild intellectual disability, chorea or other hyperkinetic movement disorders, and tongue biting. Although peripheral acanthocytes were absent, two novel pathogenic *VPS13A* mutations confirmed the diagnosis. GPi-DBS produced marked improvement in orofacial and limb movements, leading to notable functional recovery.

## CRediT authorship contribution statement

**Mitsuyoshi Tamura:** Writing – original draft, Investigation, Data curation, Conceptualization. **Shogo Furukawa:** Writing – review & editing. **Masahiro Namiki:** Writing – review & editing, Investigation. **Yukiko Ozawa:** Writing – review & editing, Investigation. **Yuka Urata:** Resources, Investigation, Formal analysis. **Masayuki Nakamura:** Supervision, Resources, Investigation, Formal analysis. **Mitsuru Watanabe:** Resources, Investigation. **Hideki Oshima:** Resources, Investigation. **Toshiki Obuchi:** Resources, Investigation. **Tadashi Ichikawa:** Supervision, Resources. **Yuriko Kikkawa:** Writing – review & editing, Supervision, Conceptualization.

## Funding

This research received no specific grant from any funding agency in the public, commercial, or not-for-profit sectors.

Data availability statement.

The data that support the findings of this study are available from the corresponding author upon reasonable request.

Consent for publication

Written informed consent for publication of this case report and accompanying images was obtained from the patient. A copy of the written consent is available for review by the Editor of this journal.

## Declaration of competing interest

The authors declare that they have no known competing financial interests or personal relationships that could have appeared to influence the work reported in this paper.
